# An invasive adenocarcinoma of the accessory parotid gland: a rare example developing from a low-grade cribriform cystadenocarcinoma?

**DOI:** 10.1186/1746-1596-6-122

**Published:** 2011-12-07

**Authors:** Shin-ichi Nakatsuka, Hiroshi Harada, Hiroshi Fujiyama, Koji Takeda, Koji Kitamura, Hayato Kimura, Teruaki Nagano, Mahito Ito, Yuji Asada

**Affiliations:** 1 Department of Pathology, Kansai Rosai Hospital, 3-1-69 Inabaso, Amagasaki, Hyogo 660-8511, Japan; 2 Department of Pathology and Research, Sakai Municipal Hospital, 1-1-1 Minami-Yasui-cho, Sakai-ku, Sakai, Osaka 590-0064, Japan; 3 Department of Plastic Reconstructive Surgery, Kansai Rosai Hospital, 3-1-69 Inabaso, Amagasaki, Hyogo 660-8511, Japan; 4 Department of Otorhinolaryngology, Kansai Rosai Hospital, 3-1-69 Inabaso, Amagasaki, Hyogo 660-8511, Japan

**Keywords:** accessory parotid gland, low-grade cribriform cystadenocarcinoma, adenocarcinoma, not otherwise specified, salivary duct carcinoma, S-100

## Abstract

Low-grade cribriform cystadenocarcinoma (LGCCA) is a rare tumor of the salivary gland that exhibits clinically indolent behavior. In this paper, we present a case of invasive adenocarcinoma of the accessory parotid gland in a young male that exhibited histology suggestive of an association of LGCCA. A 27-year-old man presented with a subcutaneous tumor in his left cheek. The tumor was separated from the parotid gland and located on the masseter muscle. The tumor was resected, and the postoperative histological diagnosis was adenocarcinoma, not otherwise specified (ANOS). The tumor exhibited papillary-cystic and cribriform proliferation of the duct epithelium and obvious stromal infiltration. Some tumor nests were rimmed by myoepithelium positive for smooth muscle actin, p63, and cytokeratin 14, indicating the presence of intraductal components of the tumor. Tumor cells exhibited mild nuclear atypia, and some of them presented an apocrine-like appearance and had cytoplasmic PAS-positive/diastase-resistant granules and hemosiderin. Other cells had foamy cytoplasm with microvacuoles. Immunohistochemistry revealed that the almost all of the tumor cells were strongly positive for S-100. These histological findings suggest the possibility that ANOS might arise secondarily from LGCCA. This is an interesting case regarding the association between ANOS and LGCCA in oncogenesis.

**Virtual Slides:**

The virtual slide(s) for this article can be found here: http://www.diagnosticpathology.diagnomx.eu/vs/1226764594634693.

## Background

Delgado *et al *originally described low-grade cribriform cystadenocarcinoma (LGCCA) as a rare low-grade variant of salivary duct carcinoma (SDC) in 1996 [1]. This tumor predominantly consists of intraductal components of the tumor and frequently exhibits papillary-cystic or cribriform proliferation similar to a low-grade ductal carcinoma *in situ *or atypical ductal hyperplasia of the breast in its histology and biologically indolent features [1-9]. This low-grade variant of SDC makes a contrast with conventional SDC, which is a clinically aggressive tumor that exhibits high-grade histology similar to an invasive ductal carcinoma of the breast [10,11]. Evidence for distinct relationships between these 2 entities has not been demonstrated; therefore, this low-grade variant of SDC is categorized as a variant of cystadenocarcinoma, termed LGCCA, in the World Health Organization classification (2005) due to its cystic morphology [2]. Past literatures have described rare cases with LGCCA that subsequently exhibited overt invasive growth in their clinical courses [6]. Herein, we present a case of invasive adenocarcinoma of the accessory parotid gland in a young male that had left vestiges of LGCCA in its histology. The invasive component of the tumor was histologically defined as adenocarcinoma, not otherwise specified (ANOS). This is an interesting case suggests that ANOS could secondarily arise from LGCCA of the salivary gland.

## Case Presentation

### Clinical summary

**Figure 1:**
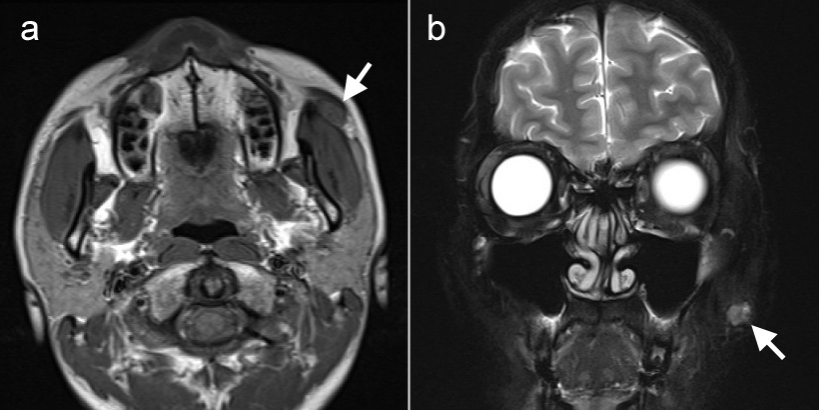
**Magnetic resonance imaging of the tumor**. The tumor is located on the left masseter muscle (arrows). **a**. Horizontal plane. The tumor exhibits hypointensity on T1-weighted images. **b**. Coronal plane. The tumor exhibits slightly higher intensity in T2-weighted imaging with a fat-suppression technique.

A 27-year-old man with more than 1-year history of a subcutaneous tumor in his left cheek consulted the Department of Plastic Reconstructive Surgery at our hospital. His past medical history and family history were unremarkable. Physical examination revealed an elastic hard tumor in the subcutis of his left cheek. The tumor did not adhere to the skin. There was no remarkable abnormality in his oral and nasal cavities. No lymph node swelling was observed in his head and neck. The patient exhibited no neurological deficits. Laboratory data were within normal limits. Magnetic resonance imaging (MRI) revealed a tumor 15 mm × 7 mm in diameter located anterior to the left masseter muscle, with lower intensity than the muscle on both T1- and T2-weighted images (Figure 1a). Imaging with a fat-suppression technique revealed slightly higher intensity in the tumor (Figure 1b). MRI findings were suggestive of a granuloma or a fibroma. The patient underwent local excision of the tumor under regional anesthesia. Grossly a whitish tumor was encapsulated by thin fibrous tissue, and it adhered to the masseter muscle. Postoperative investigation using MRI and positron emission tomography revealed no residual tumor or lymph node metastasis. The final histological diagnosis was ANOS of the accessory parotid gland. As the tumor was close to the surgical margin, we recommended that the patient receives adjuvant radiotherapy, but he did not wish to undergo the therapy. He is alive without recurrence of the disease 3 months after the surgery.

### Histological findings

**Figure 2:**
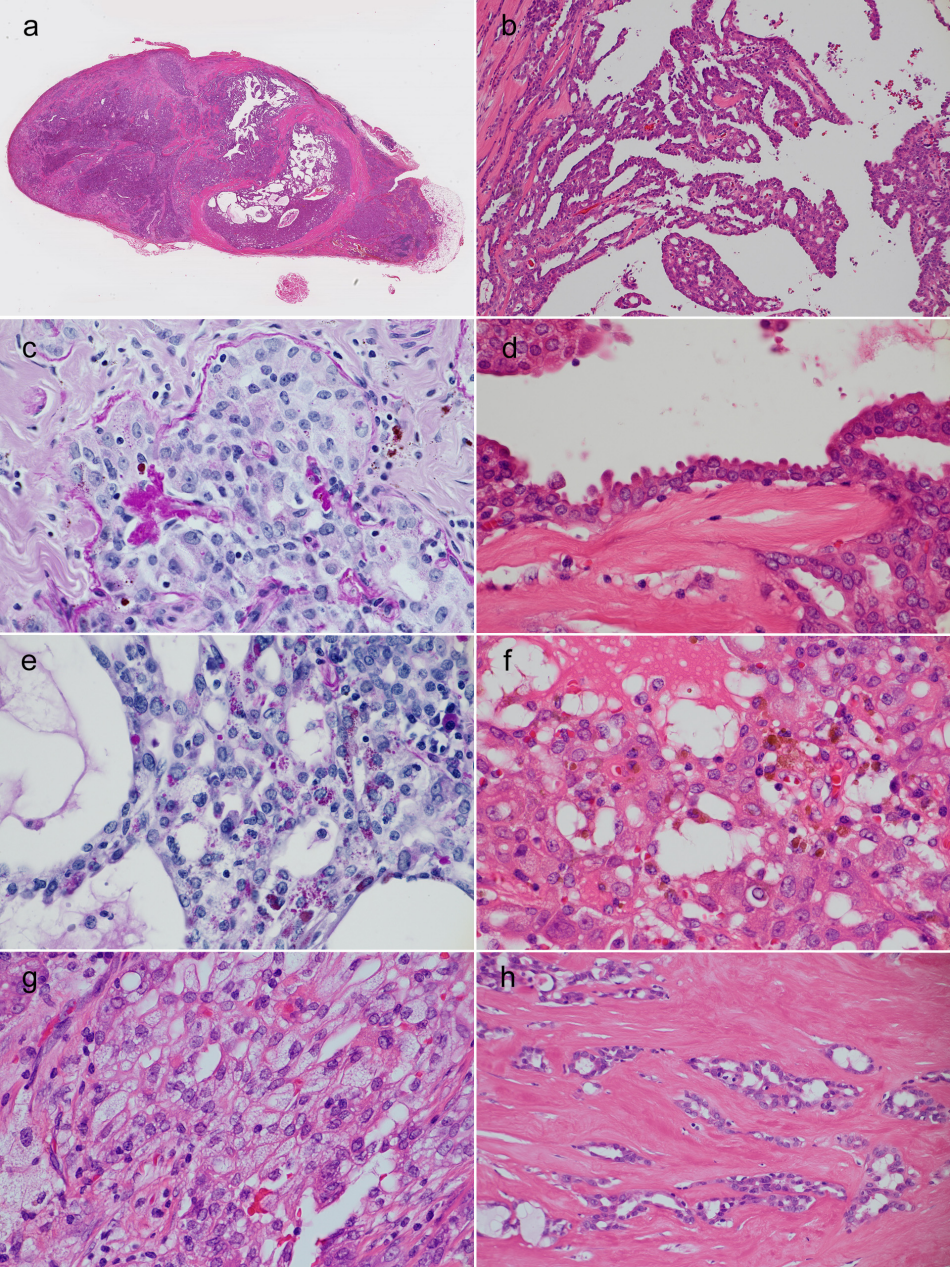
**Histological findings of the tumor**. **a**. The tumor was encapsulated but extracapsular invasion was partially observed. Papillary-cystic proliferation was observed within the tumor. Hematoxylin eosin (HE) stain, loupe. **b**. The tumor cells exhibit markedly papillary proliferation with a cribriform pattern. Comedonecrosis is not observed. HE stain, original magnification ×100. **c**. Thickened basement membranes were observed around the tumor cell nest. PAS reaction, ×400. **d**. Cuboidal tumor cells lining cystic spaces have eosinophilic cytoplasm and exhibit an apocrine-like appearance with decapitation. HE stain, ×400. **e**. Apocrine-like tumor cells have cytoplasmic PAS-positive/diastase-resistant granules. PAS reaction with diastase digestion, ×400. **f**. The tumor cells phagocytose hemosiderin in the vicinity of hemorrhage. HE stain, ×400. **g**. Some tumor cells have foamy cytoplasm similar to sebaceous cells. HE stain, ×400. **h**. Cords and tubules of the tumor infiltrate the stroma with sclerosis. HE stain, ×200.

The tumor was encapsulated by fibrous tissue but extracapsular invasion was partially observed. Normal salivary gland tissue was observed adjacent to the capsule of the tumor. It was considered an accessory parotid gland. Approximately half of the tumor consisted of irregular-shaped cystic spaces of variable size containing exudates and hemorrhage (Figure 2a). Colloid-like material such as that observed in acinic cell carcinoma was not seen. The epithelial cells lining the cyst exhibited marked papillary proliferation with a partial cribriform structure (Figure 2b). The cribriform structure included true lumens but not pseudo-lumens. Thickened basement membranes were observed around the tumor cell nests in the periodic acid-Schiff (PAS) reaction (Figure 2c). Most tumor cells had a small, round, bland nucleus with inconspicuous small nucleoli and eosinophilic cuboidal cytoplasm. An apocrine-like appearance with apical snouts was observed in some cells (Figure 2d). Apocrine-like cells had cytoplasmic PAS-positive/diastase-resistant eosinophilic granules (Figure 2e). In the vicinity of hemorrhage, tumor cells phagocytosed the brownish pigment, hemosiderin (Figure 2f). Some tumor cells had foamy cytoplasm with microvacuoles similar to those of sebaceous cells (Figure 2g). These histological features were suggestive of LGCCA, but, in the latter half of the tumor, neoplastic epithelial cords and tubules considerably infiltrated the parenchyma with myxoid stromal reactions and sclerosis (Figure 2h). This invasive component lacked specific histological features of any other salivary carcinoma; therefore, the final diagnosis of ANOS was made. Neither intravascular infiltration nor perineural infiltration was observed.

**Figure 3:**
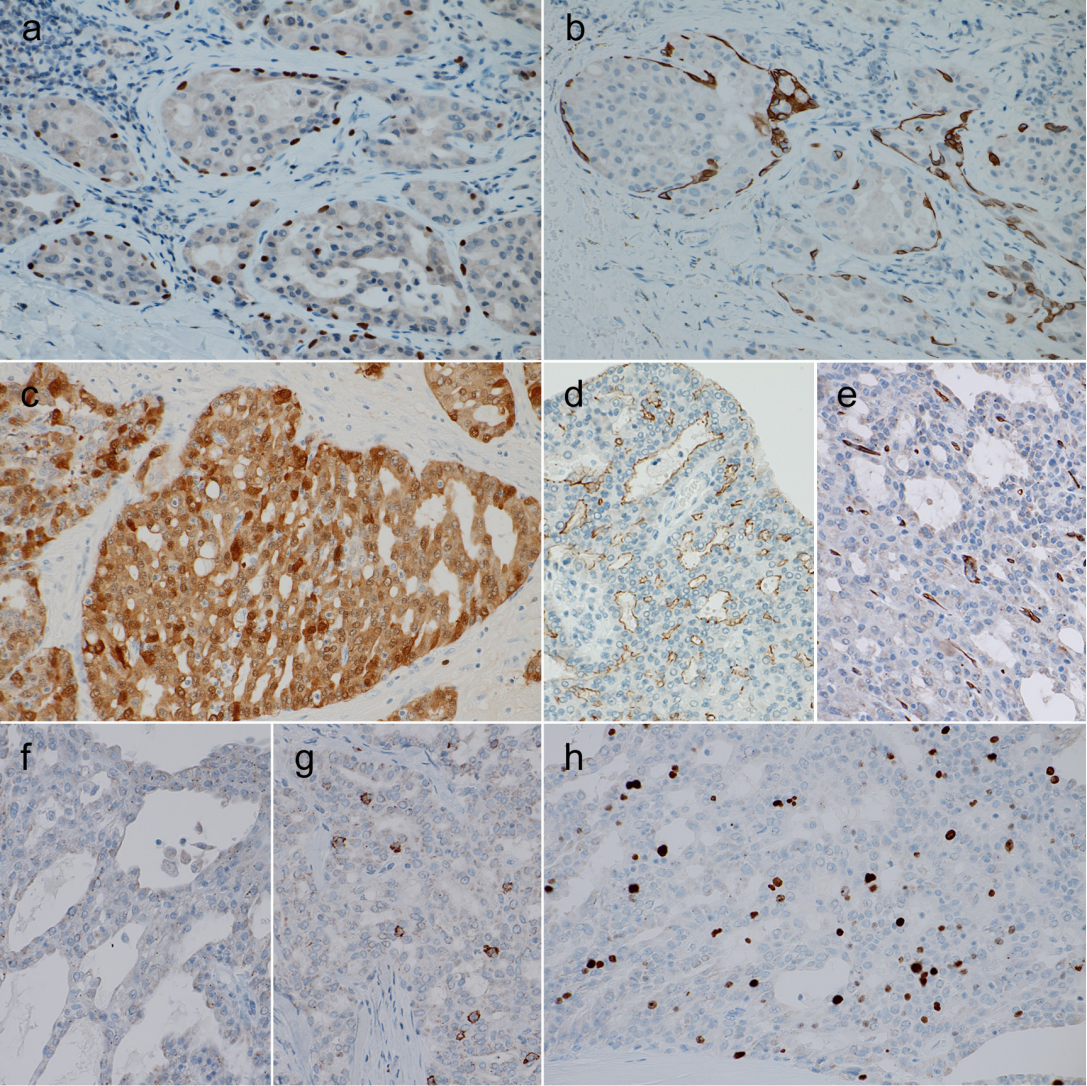
**Immunohistochemical findings of the tumor**. **a **and **b**. Myoepithelium rimming papillary-cystic structure of the tumor is positive for p63 (a) and cytokeratin 14 (b). Original magnification ×200. **c**. The tumor cells exhibit strong positivity for S-100. ×200. **d**. Carcinoembryonic antigen is positive in the lumen of cystic spaces and tubules. ×200. **e**. and **f**. The tumor cells did not exhibit positivity for neither CD34 (e) nor synaptophysin (f). ×200. **g**. CD56-positive cells were scattered. ×200. **h**. The Ki-67 labeling index was approximately 5%. ×200.

In immunohistochemistry, some of the papillary-cystic structures exhibited rimming of the myoepithelium that was positive for smooth muscle actin (SMA), p63 (Figure 3a), and cytokeratin (CK) 14 (Figure 3b), indicating the presence of intraductal neoplastic components. Tumor cells exhibited strong positivity for CK (AE1/AE3), CK 7 and S-100 (Figure 3c), which was compatible with LGCCA. Epithelial membrane antigen was partially positive. Glial fibrillary acidic protein was faintly positive in the cytoplasm. Carcinoembryonic antigen was positive in the lumen of cystic spaces and tubules (Figure 3d). CK 20, gross cystic disease fluid protein (GCDFP)-15, CD34 (Figure 3e), and p53 were negative. SMA, p63, and CK 14 were negative in the neoplastic cells other than the myoepithelium rimming the cell nest. As neuroendocrine markers, synaptophysin was negative (Figure 3f) and a small number of CD56-positive cells were scattered (Figure 3g). Intracytoplasmic granules exhibited faintly non-specific staining with chromogranin A. The Ki-67 labeling index was approximately 5% (Figure 3h).

## Conclusions

The tumor of the present case was grossly separated from the parotid gland. As the tumor was located on the masseter muscle and a histologically normal salivary gland was observed in the periphery of the tumor, the tumor might have developed from the accessory parotid gland. An accessory salivary gland is occasionally observed on the masseter muscle along with Stensen's duct. Neoplasms arising from an accessory parotid gland are relatively rare, and variable types of malignant tumor have been reported in the literature: carcinoma ex pleomorphic adenoma [12], squamous cell carcinoma [13], mucoepidermoid carcinoma [14], acinic cell carcinoma [15], oncocytic carcinoma [16], basal cell carcinoma [14], and small cell carcinoma [14].

The tumor of the present case included a component exhibiting papillary-cystic proliferation and another component with obvious invasion to the parenchyma with a tubular or scirrhous pattern. Since the invasive component of the tumor did not exhibit specific histological features of any other defined carcinoma, we made the diagnosis of ANOS. There was myoepithelial rimming of the tumor cell nest with positivity for SMA, p63, and CK14 in some areas within the tumor, indicating intraductal proliferation of the duct epithelium. Additionally, the PAS reaction clearly revealed thickened basement membranes around the tumor cell nests. These findings allowed the presumption that the present tumor might have developed in the precedent intraductal tumor. In addition, the tumor cells presented an apocrine-like appearance and included PAS-positive/diastase-resistant granules and hemosiderin in the cytoplasm, in line with findings frequently observed in LGCCA [1-9]. Strong positivity of S-100 in immunohistochemistry in almost all tumor cells was also characteristic of LGCCA. The aforementioned histological findings indicated the presence of histological vestiges of LGCCA in this tumor, suggesting the possibility that ANOS in this case might have arise secondarily from the tumor initially developing as LGCCA in the salivary gland. In principle, LGCCA has good clinical behavior and exhibits neither recurrence nor metastasis [1-9], although Weinreb *et al *reported a rare case of a low-grade intraductal carcinoma of the parotid gland that subsequently progressed to adenosquamous carcinoma [6]. Ihrler *et al *revealed that an intraductal component was identified in 15 of 22 patients (68%) with ANOS and speculated that the intraductal tumor is identified as the preinvasive precursor of ANOS [17]. Although there has never been a description of the association between ANOS and LGCCA, the present case suggested the possibility that a certain group of ANOS could develop secondarily from LGCCA. To verify this speculation, accumulation of the evidence about ANOS and LGCCA using molecular biology techniques will be required hereafter.

One of the most important differential diagnoses of the present case is a papillary cystic variant of acinic cell carcinoma (PCV-ACC). PCV-ACC preferentially affects young people [18]. Tumor cells of PCV-ACC have intracytoplasmic PAS-positive/diastase-resistant granules (zymogen) and hemosiderin, as observed in LGCCA [18]. However, PCV-ACC is typically negative for S-100 and typically does not exhibit predominance of the intraductal component of the tumor in histology. Sebaceous cell-like foamy cells with microvacuoles are sometimes seen in cystadenocarcinoma of the salivary gland. Intracytoplasmic vacuoles are found also in PCV-ACC, but uniformly-sized, fine microvacuoles might be considered characteristic of cystadenocarcinomas, including LGCCA, rather than PCV-ACC [19]. Another differential diagnosis is SDC. SDC is a high-grade adenocarcinoma that is common in elderly people over 50 years of age [10,11]. Histologically, SDC resembles a high-grade invasive ductal carcinoma of the breast, frequently accompanied by comedonecrosis and cribriform proliferation [10,11]. SDC exhibits an apocrine-like appearance with positivity for GCDFP-15 and androgen receptor, which is occasionally observed in LGCCA, but SDC is negative for S-100. SDC usually exhibits a high Ki-67 labeling index, whereas the index in our case was low (approximately 5%). Pleomorphic adenoma and carcinoma ex pleomorphic adenoma should be listed as a differential diagnosis [20], although the tumor of our case did not include a component showing typical histology of pleomorphic adenoma, such as transition from myoepithelium to stromal spindle cell and myxoid or cartilaginous stroma. Neuroendocrine tumors were excluded because rosette-like or trabecular configuration was not observed and almost all tumor cells did not express neuroendocrine markers except for scattered CD56-positive cells.

We presented a case of ANOS developing in the accessory salivary gland that suggests an association with LGCCA. This is an interesting case when considering the relationship between ANOS and LGCCA in oncogenesis. This case is meaningful in reconsidering the disease entity and the process of development of ANOS of the salivary gland.

## Consent

Written informed consent was obtained from the patient for publication of this Case Report and any accompanying images. A copy of the written consent is available for review by the Editor-in-Chief of this journal.

## List of abbreviations

ANOS: adenocarcinoma, not otherwise specified; CK: cytokeratin; GCDFP: gross cystic disease fluid protein; LGCCA: low-grade cribriform cystadenocarcinoma; PCV-ACC: papillary cystic variant of acinic cell carcinoma; SDC: salivary duct carcinoma; SMA: smooth muscle actin.

## Competing interests

The authors declare that they have no competing interests.

## Authors' contributions

SN was responsible for literature search and manuscript preparation. HH participated in the discussion for histological diagnosis and manuscript preparation. HF, KT and YA collected the clinical data and performed the surgery. KK and MI performed postoperative clinical follow-up of the patient. TN and HK participated in the microscopic analyses. All authors read and approved the final manuscript.
